# Characterization of gene expression profiles in the mouse brain after 35 days of spaceflight mission

**DOI:** 10.1038/s41526-022-00217-4

**Published:** 2022-08-10

**Authors:** Jacob M. Holley, Seta Stanbouly, Michael J. Pecaut, Jeffrey S. Willey, Michael Delp, Xiao Wen Mao

**Affiliations:** 1 Department of Basic Sciences, Division of Biomedical Engineering Sciences (BMES), Loma Linda University School of Medicine, Loma Linda, CA 92350 USA; 2 Department of Radiation Oncology, Wake Forest University, School of Medicine, Winston-Salem, NC 27101 USA; 3 Department of Nutrition, Food and Exercise Sciences, Florida State University, Tallahassee, FL 32306 USA

**Keywords:** Neuroscience, Chronic inflammation

## Abstract

It has been proposed that neuroinflammatory response plays an important role in the neurovascular remodeling in the brain after stress. The goal of the present study was to characterize changes in the gene expression profiles associated with neuroinflammation, neuronal function, metabolism and stress in mouse brain tissue. Ten-week old male C57BL/6 mice were launched to the International Space Station (ISS) on SpaceX-12 for a 35-day mission. Within 38 ± 4 h of splashdown, mice were returned to Earth alive. Brain tissues were collected for analysis. A novel digital color-coded barcode counting technology (NanoString^TM^) was used to evaluate gene expression profiles in the spaceflight mouse brain. A set of 54 differently expressed genes (*p* < 0.05) significantly segregates the habitat ground control (GC) group from flight (FLT) group. Many pathways associated with cellular stress, inflammation, apoptosis, and metabolism were significantly altered by flight conditions. A decrease in the expression of genes important for oligodendrocyte differentiation and myelin sheath maintenance was observed. Moreover, mRNA expression of many genes related to anti-viral signaling, reactive oxygen species (ROS) generation, and bacterial immune response were significantly downregulated. Here we report that significantly altered immune reactions may be closely associated with spaceflight-induced stress responses and have an impact on the neuronal function.

## Introduction

The spaceflight environment is characterized mainly by ultraviolet and ionizing radiation, microgravity, and physiological/psychological stressors. These conditions present a significant hazard to spaceflight crews during the course of mission activities. The susceptibility of the central nervous system (CNS) to spaceflight-induced changes can be particularly devastating to the health, mission performance, and quality of life of the spaceflight crew both acutely and chronically. The health risk from spaceflight-induced neuronal damage and potential adverse neurovascular effects are a chief concern, examples are intracranial fluid redistribution results in alteration of brain perfusion, neurovestibular problems, and cognitive alterations in astronauts^1,2^ caused by microgravity and/or strong gravitational changes during ascent and re-entry. Long-duration spaceflights reportedly induce immune dysregulation, which is considered a risk to astronaut safety and mission success^3^. Studies to date have demonstrated the altered distribution of peripheral leukocytes, a diminished function of specific leukocyte subpopulations, and skewed cytokine profiles in many astronauts^3^. Many biomarkers associated with neuroinflammation following space radiation and space stressors in rodents and humans have been identified^4–8^. However, the precise nature of immune dysregulation during spaceflight is not well understood. Evidence suggests that acute exposure to galactic cosmic rays (GCRs) induce detrimental CNS changes including an increased level of neuroinflammation, neuronal damage and cognitive deficits similar to accelerated aging^9–12^. Ionizing radiation has been shown to elicit neuroinflammation through direct activation of microglia cells^13^ and through the increased infiltration of immune and inflammatory cells through the damaged blood-brain barrier (BBB)^14^. Study also showed that both microgravity encountered by astronauts in space and simulated microgravity on earth induce changes in brain structure and function^15^. Microgravity induces a tremendous shift in body fluids, an increase in brain fluid and alterations in tissue perfusion^16^. Space microgravity modulates the expression of cellular molecules, and alters pro-inflammation cytokine secretion^17^. Neuroinflammation is also a central pathological feature of several acute and chronic brain diseases, including Alzheimer’s disease (AD), Parkinson disease, amyotrophic lateral sclerosis, and multiple sclerosis^18^.

The CNS is sensitive to oxidative injury due to its high oxygen consumption^19^, the content of oxidizable unsaturated lipids^20^ and low levels of anti-oxidant defenses^21^. Oxidative injury has been implicated as a causative or contributory factor in a number of neurodegenerative conditions, including aging, and ischemic, traumatic damage^22–26^. Our preliminary studies have shown that spaceflight and ionizing radiation cause prolonged oxidative stress and endothelial dysfunction that may lead to chronic inflammation and adverse remodeling^27–30^. However, our knowledge about the mechanism and consequences of spaceflight condition-induced neuroinflammation is very limited. Because of the complexity of the processes and pathways that lead to neuroinflammation, an in-depth “omics” approach will potentially provide insight to understanding the impact of environmental insults on the CNS and the immune function that may have a long-lasting impact.

The goal of this study was to characterize changes in gene expression using a neuroinflammatory assay panel to investigate inflammation, neuronal function, growth, metabolism, and stress in mouse brain tissue after spaceflight.

## Results

### Changes of gene expression in neuroinflammation panel

A set of 54 differently expressed genes (DEG) significantly (*p* < 0.05) by one-way analysis of variance (ANOVA) and Tukey’s HSD (honestly significant difference) test segregates the ground control (GC) group from the flight (FLT) group (Table 1). Clusters of genes related to neuronal function, neuronal cell support, immune function, and cellular growth and stress were significantly altered and organized based on their protein’s role. SIN3A (https://www.ncbi.nlm.nih.gov/gene/?term=NM_001110350), SLC2A5 (https://www.ncbi.nlm.nih.gov/gene/?term=NM_019741), MERTK (https://www.ncbi.nlm.nih.gov/gene/?term=NM_008587), TREM2 (https://www.ncbi.nlm.nih.gov/gene/?term=NM_025864), RIPK1 (https://www.ncbi.nlm.nih.gov/gene/?term=NM_009068) are listed in multiple tables as they are found to play various roles.

**Table 1:** Summary of the 54 differentially expressed genes in flight (FLT) mice brains relative to habitat ground controls (GC).

Gene name (mRNA)	Functions	Log2 fold Δ	LC limit (log2)	UC limit (log2)	*p* value
Optn	Autophagy, cell cycle, microglia function	−0.435	−0.547	−0.324	1.72E−05
Mertk	Autophagy, microglia function	−0.368	−0.499	−0.237	0.000256
Cd74	Adaptive immune response, inflammatory signaling	−1	−1.45	−0.548	0.00146
Plp1	Oligodendrocyte function	−0.363	−0.54	−0.187	0.00238
Sox4	Microglia function, Wnt	0.314	0.156	0.473	0.00304
H2-T23	Adaptive immune response, astrocyte function, inflammatory signaling, innate immune response, matrix remodeling	0.357	0.165	0.548	0.00447
Irf7	Apoptosis, inflammatory signaling, innate immune response	−1.06	−1.63	−0.487	0.00544
Reln	Growth factor signaling, matrix remodeling	−0.535	−0.836	−0.235	0.00583
Prkar2b	Apoptosis, cell cycle, growth factor signaling	−0.32	−0.503	−0.137	0.00641
Agt	Astrocyte function	0.432	0.184	0.679	0.00657
Aldh1l1	Astrocyte function	−0.345	−0.542	−0.147	0.00659
Brd2	Epigenetic regulation	−0.182	−0.286	−0.0773	0.00667
Ccng2	Cell cycle, DNA damage	0.292	0.119	0.465	0.00785
Hspb1	Angiogenesis, astrocyte function, cellular stress, growth factor	0.706	0.284	1.13	0.00825
Dlg4	Adaptive immune response, angiogenesis, cytokine signaling, growth factor signaling, neurons and neurotransmission	−0.279	−0.45	−0.108	0.00947
Cd6	Matrix remodeling	−0.972	−1.56	−0.388	0.00978
Csk	Adaptive immune response, angiogenesis, growth factor	−0.236	−0.384	−0.0878	0.0108
F3	Microglia function	−0.304	−0.496	−0.113	0.011
Csf1r	Cytokine signaling, growth factor signaling, microglia function	−0.209	−0.341	−0.0771	0.0111
Uty	Epigenetic regulation	0.308	0.113	0.502	0.0112
Kcnd1	Microglia function, neurons and neurotransmission	−0.893	−1.45	−0.337	0.0117
Cyp27a1	Inflammatory signaling	−0.805	−1.32	−0.292	0.0132
Slc2a5	Microglia function	−0.623	−1.02	−0.226	0.0132
Sin3a	Epigenetic regulation	−0.43	−0.715	−0.146	0.0142
E2f1	Apoptosis, cell cycle, cellular stress, notch	−0.709	−1.17	−0.25	0.0143
Egfr	Adaptive immune response, angiogenesis, astrocyte function	−0.509	−0.841	−0.177	0.0149
Tmem206	Microglia function	−0.239	−0.407	−0.0701	0.0196
C5ar1	Inflammatory signaling, neurons and neurotransmission	−0.851	−1.44	−0.259	0.0201
Opalin	Oligodendrocyte function	−0.546	−0.935	−0.157	0.0205
Ncf1	Adaptive immune response, angiogenesis, cellular stress	−0.539	−0.918	−0.159	0.0214
Mre11a	DNA damage	−0.383	−0.665	−0.1	0.0241
Ikbkg	Adaptive immune response, apoptosis, inflammatory signaling	−0.27	−0.47	−0.0697	0.0246
Prkaca	Adaptive immune response, angiogenesis, apoptosis, Wnt	−0.144	−0.251	−0.0371	0.0248
Apoe	Astrocyte function, cellular stress, lipid metabolism, microglia	0.296	0.072	0.521	0.027
St8sia6	Microglia function	−0.479	−0.835	−0.123	0.0272
Gpr62	Oligodendrocyte function	−0.418	−0.736	−0.1	0.0276
Hpgds	Lipid metabolism	−0.527	−0.922	−0.132	0.0282
Sesn1	DNA damage	0.226	0.0509	0.4	0.0298
Cdc25a	Cell cycle, DNA damage	0.218	0.0481	0.389	0.0307
Lmna	Apoptosis, cell cycle, microglia function	−0.284	−0.507	−0.0596	0.0324
Map2k4	Adaptive immune response, apoptosis, cellular stress	−0.2	−0.359	−0.041	0.0334
Irf8	Inflammatory signaling, microglia function	−0.51	−0.914	−0.106	0.0352
Trem2	Adaptive immune response, inflammatory signaling, microglia	−0.461	−0.833	−0.088	0.0359
Mpeg1	Inflammatory signaling	−0.292	−0.528	−0.0551	0.0363
Rhoa	Angiogenesis, autophagy, growth factor signaling, Wnt	0.196	0.0351	0.357	0.0381
Tarbp2	Epigenetic regulation	0.195	0.0346	0.355	0.0384
Ripk1	Apoptosis, innate immune response, NF-kB	−0.63	−1.14	−0.119	0.0389
Gadd45g	Cell cycle, DNA damage, growth factor signaling	0.392	0.0686	0.716	0.0389
Brd3	Epigenetic regulation	−0.305	−0.557	−0.0526	0.0394
Nrgn	Neurons and neurotransmission	−0.16	−0.295	−0.0261	0.0413
Nlgn2	Matrix remodeling, neurons and neurotransmission	−0.306	−0.562	−0.0486	0.042
Bbc3	Apoptosis, DNA damage	−0.538	−0.987	−0.0897	0.0432
Rgl1	Growth factor signaling, microglia function	0.105	0.0146	0.196	0.0462
Creb1	Adaptive immune response, carbohydrate metabolism, DNA damage, innate immune response, notch	0.235	0.0291	0.44	0.0493

*LC* lower confidence, *UC* upper confidence.

### Changes of gene expression associated with neuronal function

**Fig. 1:**
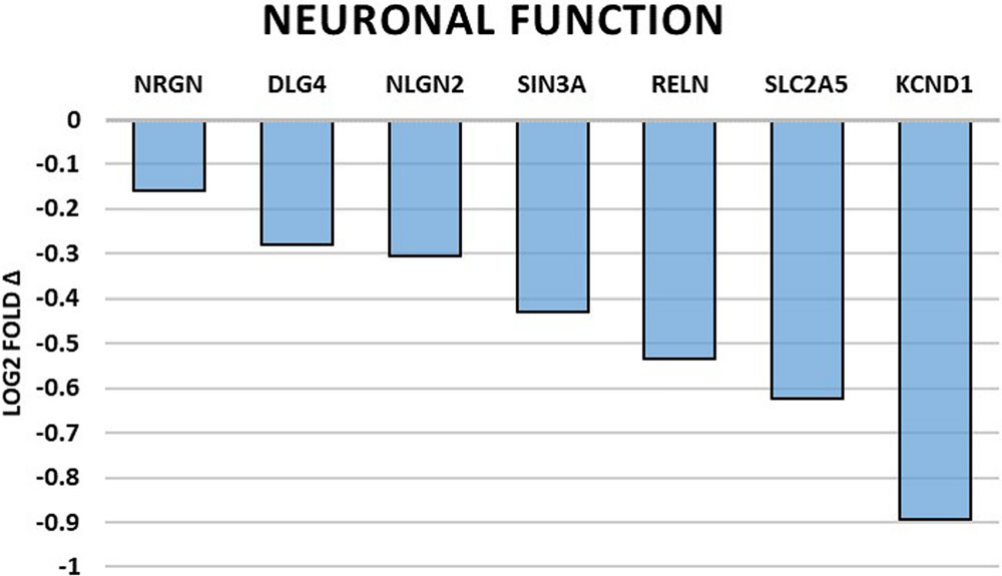
Spaceflight-induced changes of gene expression related to neuronal function. Bar graph summarizing log2 fold-changes of significantly differentially expressed genes (DEG) (*p* < 0.05) in the flight (FLT) group compared to the ground control (GC) group in genes directly related to neuronal function. *N* = 6/group. *p* values are calculated using one-way analysis of variance (ANOVA) and Tukey’s HSD (honestly significant difference) test. Source data are provided as a Source Data file.

**Fig. 2:**
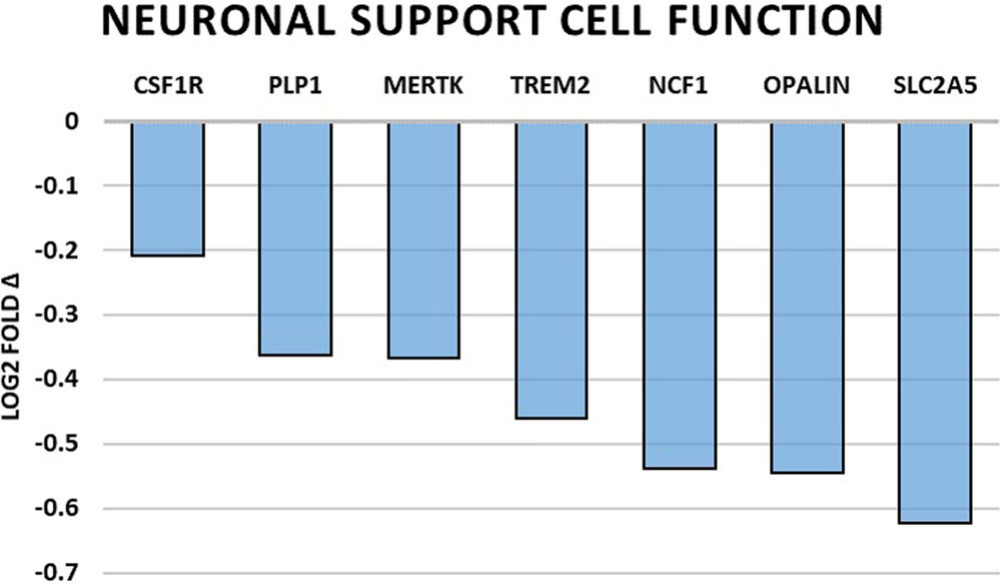
Spaceflight-induced changes of gene expression related to neuronal support cell function. Bar graph summarizing log2 fold-changes of significantly differentially expressed genes (DEG) (*p* < 0.05) in the flight (FLT) group compared to the ground control (GC) group in genes directly related to neuronal supporting cell function. N = 6/group. *P* values are calculated using one-way analysis of variance (ANOVA) and Tukey’s HSD (honestly significant difference) test. Source data are provided as a Source Data file.

**Fig. 3:**
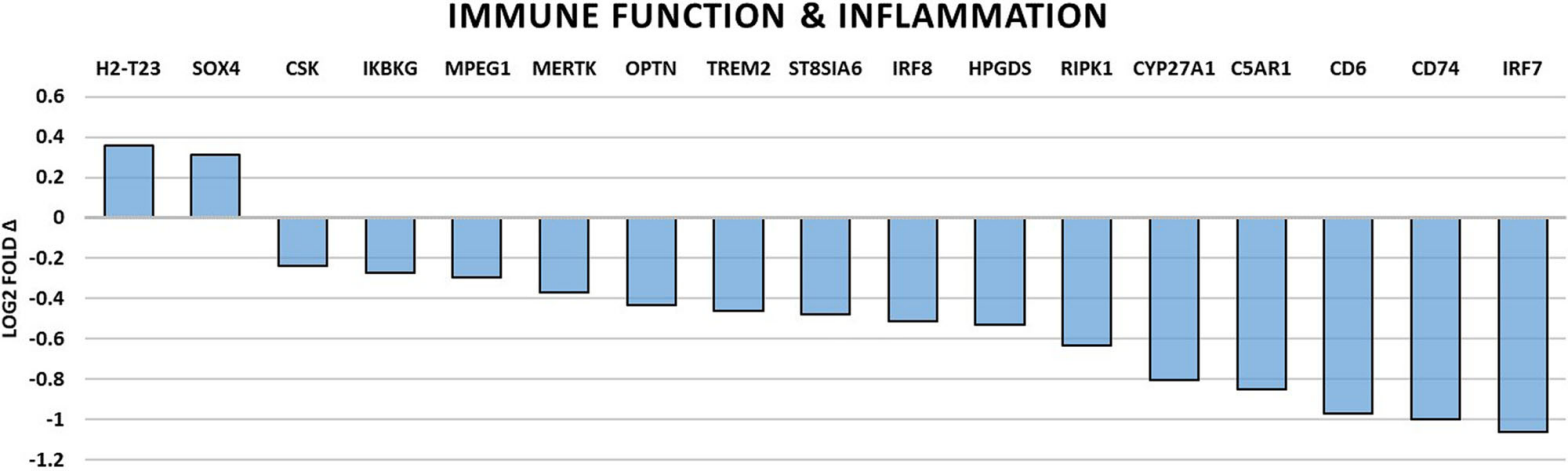
Spaceflight-induced changes of gene expression related to immune function and inflammation. Bar graph summarizing log2 fold-changes of significantly differentially expressed genes (DEG) (*p* < 0.05) in the flight (FLT) group compared to the ground control (GC) group in genes directly related to immune function and inflammation. *N* = 6/group. *p* values are calculated using one-way analysis of variance (ANOVA) and Tukey’s HSD (honestly significant difference) test. Source data are provided as a Source Data file.

**Fig. 4:**
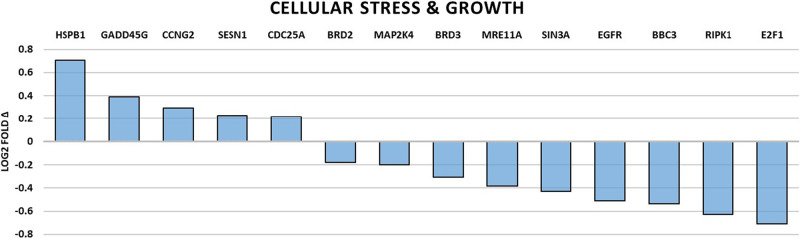
Spaceflight-induced changes of gene expression related to cellular stress and growth function. Bar graph summarizing log2 fold-changes of significantly differentially expressed genes (DEG) (*p* < 0.05) in the flight (FLT) group relative to the ground control (GC) group in genes directly related to cellular stress and growth function. *N* = 6/group. *p* values are calculated using one-way analysis of variance (ANOVA) and Tukey’s HSD (honestly significant difference) test. Source data are provided as a Source Data file.

Genes directly involved with neuronal function were universally downregulated and are summarized in Table 2 and Fig. 1. Overall, a picture of decreased neuron plasticity and signaling dysfunction is painted. Those genes that are involved in neuron support cell function were also found to be broadly downregulated and are summarized in Table 3 and Fig. 2. Microglial cell and oligodendrocyte dysfunction were most pronounced along with the inhibition of myelin sheath maintenance. Levels of immune and inflammation related gene expression were grossly downregulated except for two upregulated genes, H2-T23 (https://www.ncbi.nlm.nih.gov/gene/?term=NM_010398) and SOX4 (https://www.ncbi.nlm.nih.gov/gene/?term=NM_009238). The summary is presented in Table 4 and Fig. 3. Generally, we see the impairment of genes related to microbial defense with generalized dysfunction of immunity and disinhibition of inappropriate inflammation. And finally, genes with roles in cellular stress and growth are shown in Fig. 4 with downregulation in all genes with exception of HSPB1 (https://www.ncbi.nlm.nih.gov/gene/?term=NM_013560), GADD45G (https://www.ncbi.nlm.nih.gov/gene/?term=NM_011817), CCNG2 (https://www.ncbi.nlm.nih.gov/gene/?term=NM_007635), SESN1 (https://www.ncbi.nlm.nih.gov/gene/?term=NM_001013370), and CDC25A (https://www.ncbi.nlm.nih.gov/gene/?term=NM_007658) which were significantly upregulated. In general, we see an increased cellular stress response, growth arrest, and a possible trend toward more inflammatory-associated cell death.

**Table 2:** Summary of significantly altered gene expressions by spaceflight related to neuronal function.

Gene	*p* value	Log2 fold Δ	Related molecule function
NRGN	0.0413	−0.16	A Ca2+ dependent intracellular charge transducer.
DLG4	0.00947	−0.279	Postsynaptic density protein associated with glutamatergic receptor signaling.
NLGN2	0.042	−0.306	Postsynaptic cell adhesion molecule mediating inhibitory synapses.
SIN3A	0.0142	−0.43	Involved in cortical neuron differentiation and callosal axon elongation.
RELN	0.00583	−0.535	Regulates microtubule function in neurons and neuronal migration.
SLC2A5	0.0132	−0.623	Fructose transporter found in Purkinje cells and the blood-brain barrier.
KCND1	0.0117	−0.893	K+ voltage-gated channel involved in neurotransmitter release.

**Table 3:** Summary of significantly altered gene expressions by spaceflight related to neuronal support cell function.

Gene	*p* value	Log2 fold Δ	Related molecule function
CSF1R	0.0111	−0.209	Cell-surface receptor regulating development, proliferation and differentiation.
PLP1	0.00283	−0.363	Major myelin protein in the CNS.
MERTK	0.000256	−0.368	Signaling protein in innate immune cells mediating engulfment of apoptotic cells.
TREM2	0.0359	−0.461	Regulates microglial chemotaxis and phagocytosis of apoptotic neurons.
NCF1	0.0214	−0.539	Subunit of NADPH oxidase required for superoxidase production.
OPALIN	0.0205	−0.546	Regulator of oligodendrocyte cytoskeletal remolding and morphology.
SLC2A5	0.0132	−0.623	Fructose transporter found in microglial cells.

**Table 4:** Summary of significantly altered gene expressions by spaceflight related to immune function and inflammation.

Gene	*p* value	Log2 fold Δ	Related molecular function
H2-T23	0.00447	0.357	Immune regulation and protection through NK and T-cell suppression.
SOX4	0.00304	0.314	Transcription factor regulating key genes in Thelper17 cells.
CSK	0.0108	−0.236	Negative regulator of T-cell receptor signaling.
IKBKG	0.0246	−0.27	Subunit of IKK complex with cardinal role in stimulating NF-κB regulation.
MPEG1	0.0363	−0.292	Pore-forming bactericidal effector molecule of the innate immune system.
MERTK	0.000256	−0.368	Signaling protein in innate immunity for a negative regulator of inflammation.
OPTN	0.0000172	−0.435	Ubiquitin-binding protein important for bacterial autophagic clearance.
TREM2	0.0359	−0.461	Inhibitor of neuroinflammation via suppression of NF-kB signaling.
ST8SIA6	0.0272	−0.479	A sialyltransferase important for immune suppression and modulation.
IRF8	0.0352	−0.51	Transcription factor for anti-viral dendritic cells and IFN-inducible genes.
HPGDS	0.0282	−0.527	Enzymatic mediator of prostaglandin D2 creation.
RIPK1	0.0389	−0.63	Promotor of survival, apoptotic and inflammatory signaling pathways.
CYP27A1	0.0132	−0.805	Cytochrome P450 enzyme important in the metabolism of cholestrol.
C5AR1	0.0201	−0.852	Receptor for the chemotactic and inflammatory anaphylatoxin, C5a.
CD6	0.00978	−0.972	Stimulatory molecule promoting T-cell activation, proliferation, and adhesion.
CD74	0.00146	−1	Chaperone protein involved with MHC class II antigen presentation.
IRF7	0.00544	−1.06	Transcriptional regulator of type I interferon dependent immune responses.

### Spaceflight-induced changes of pathways

**Fig. 5:**
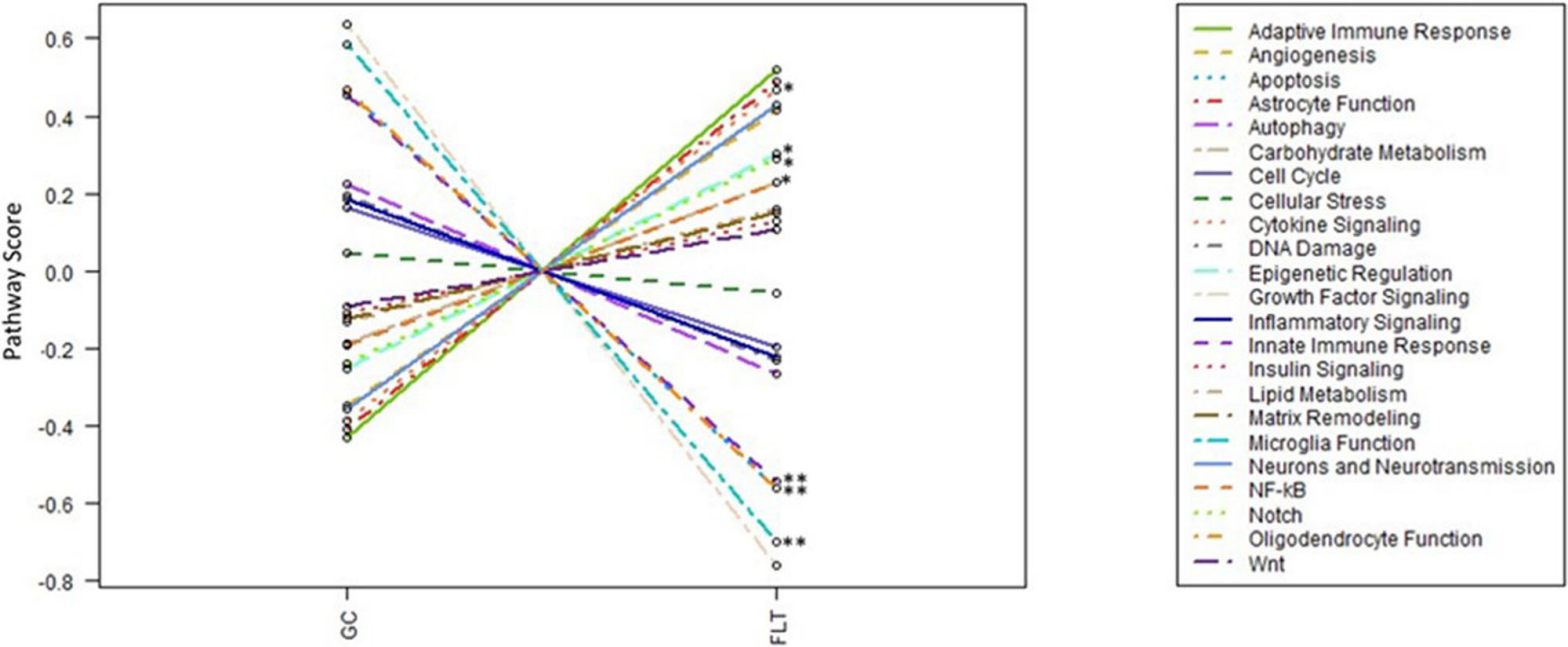
Spaceflight-induced changes of pathway scores. Summarized pathway scores in flight (FLT) group vs. ground control (GC). *Significantly upregulated pathways (*p* < 0.05), include:  cytokine signaling,  Angiogenesis,  Epigenetic regulation, and  Notch. **Significantly (*p* < 0.05) or strong trend (*p* = 0.07) downregulated pathways, include:  Oligodendrocyte function,  Innate immune response, and  Microglia function. *p* values are by one-way ANOVA and Tukey’s post hoc test. Source data are provided as a Source Data file.

**Fig. 6:**
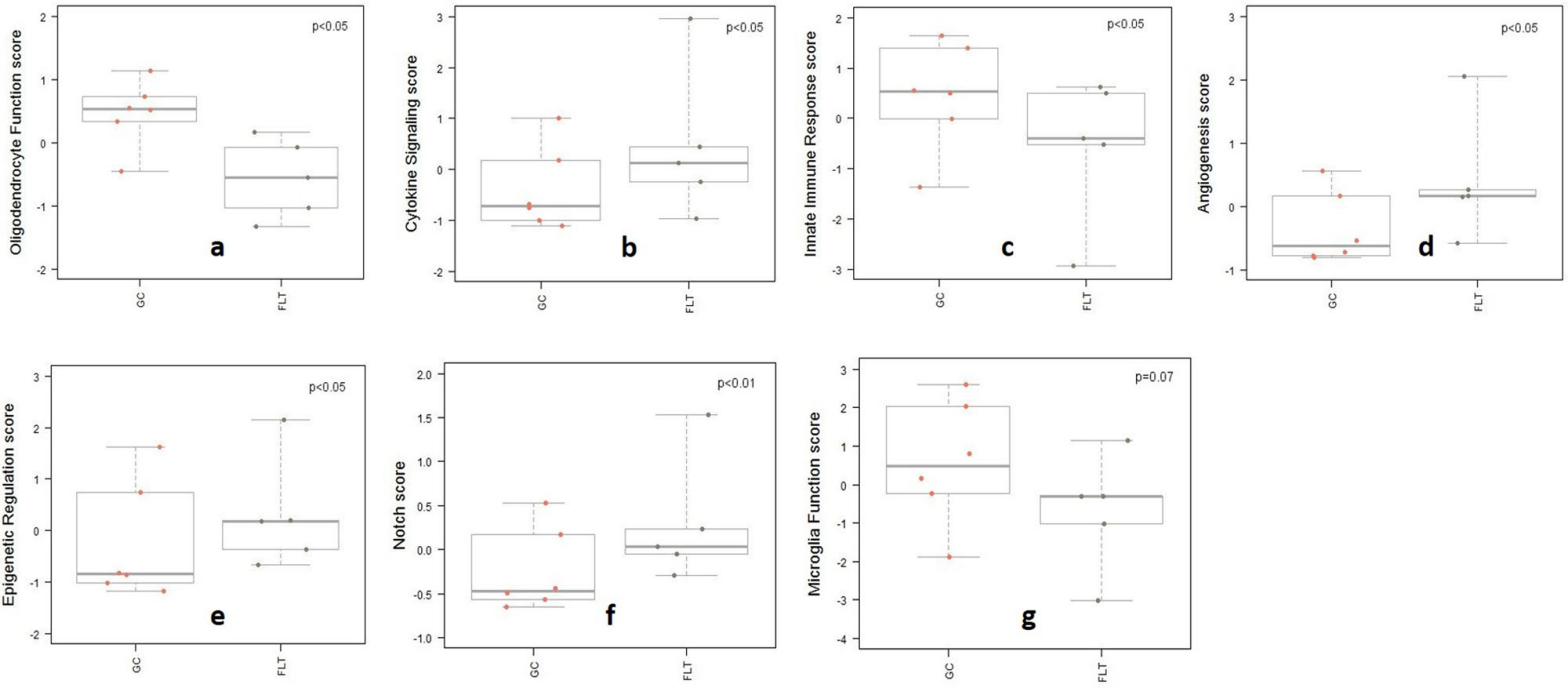
Spaceflight-induced changes in nueroinflammation pathway scores among flight (FLT) and ground controls (GC) groups. Boxplots depict pathway scores on the *y*-axis and the experimental conditions on the *x*-axis. *N* = 5–6 /group. *p* values are calculated using one-way analysis of variance (ANOVA) and Tukey’s HSD (honestly significant difference) test. **a** Oligodendrocyte function score *p* < 0.05, **b** cytokine signaling score *p* < 0.05, **c** innate immune response score *p* < 0.05, **d** angiogenesis score *p* < 0.05, **e** epigenetic regulation score *p* < 0.05, **f** notch score *p* < 0.01, and **g** Microglia function score *p* = 0.07. Boxes are the range between first (25%) and the third (75%) quartile, the center line is the median, the whiskers include the variability those quartiles. Source data are provided as a Source Data file.

Many pathways associated with cellular stress, inflammation, apoptosis, and metabolism were altered by flight condition using DEG identified by Nanostring’s Advanced Analysis module pathway scoring through correlation-guided gene subsetting, which functionally annotated groups of genes followed by unsupervised clustering of samples^31^. Higher scores compared to normalized gene expression general means upregulation while lower scores represent downregulation. Figure 5 summarized pathway results in a plot of all pathway scores compared to GC controls. Among these pathways, scores for angiogenesis, cytokine signaling, epigenetic regulation, and notch signaling were significantly increased which may indicate upregulation of the pathway (*p* < 0.05), while scores for innate immune response and oligodendrocyte function were greatly reduced in the flight group compared to the controls which may indicate downregulation of the pathways (*p* < 0.05) by one-way ANOVA and Tukey’s HSD test (Fig. 6a–f). The score for microglia function was also decreased but with trending significance (*p* = 0.07) (Fig. 6g).

## Discussion

Our data showed robust changes in gene expression profiles after spaceflight. Genes supporting neuronal synaptic signaling and migration were significantly downregulated. Decreased expression of genes important for oligodendrocyte differentiation and myelin sheath maintenance was observed. Downregulation of microglial gene expression that is important for apoptotic cell clearance, phagocytosis, and proliferation were evident. Genes supporting innate immune responses were also downregulated regarding anti-viral signaling, reactive oxygen species (ROS) generation, and bacterial immune response. Expression of genes that are correlated with DNA damage and cellular stress were upregulated. Many of these observed changes are in line with our previous findings in the brains exposed to simulated spaceflight condition. Following combined exposure of simulated microgravity and radiation, pathways involved in neurogenesis, neuroplasticity, the regulation of neuropeptides, neuronal structure, stress, and cellular signaling are significantly altered^29^. Gene expression in the brain that was analyzed after 90 days of simulated microgravity showed many genes related to catalytic and oxidoreductase activities were downregulated^32^. However, in contrary to our findings, many genes related to stress, immune response, metabolic process, and/or inflammatory response were significantly upregulated in their study. Duration of environmental stress may play a role in different response of gene expression profiles and pathway regulation.

We saw uniform downregulation of gene expression in our mouse brain samples directly related to neuron function. NRGN (https://www.ncbi.nlm.nih.gov/gene/?term=NM_022029) coding for a postsynaptic kinase called neurogranin was found to be significantly reduced in expression. When neurogranin binds calmodulin, the concentration of calcium needed to transduce a signal is increased^33^. Given the downregulation of NRGN (https://www.ncbi.nlm.nih.gov/gene/?term=NM_022029) and possible decrease in availability of neurogranin, neurons may become more sensitive to firing. KCND1(https://www.ncbi.nlm.nih.gov/gene/?term=NM_008423) codes for a potassium voltage-gated channel involved in neurotransmitter release which has been shown to be inhibited by some types of ROS generating treatments^34–36^. Environmental stressor-induced ROS may be provoking the down regulation of KCND1(https://www.ncbi.nlm.nih.gov/gene/?term=NM_008423). DLG4 (https://www.ncbi.nlm.nih.gov/gene/?term=NM_001109752) is transcribed into a protein associated with glutamatergic receptor signaling while also potentially participating in dendritogenesis^37^. Its downregulation observed in our assay has the potential to affect glutamatergic neuron signaling, and affect dendritic morphology leading to further dysfunction. NLGN2 (https://www.ncbi.nlm.nih.gov/gene/?term=NM_198862) codes for a molecule which selectively mediates inhibitory synapses^38^. Its downregulation may contribute to dysfunction at inhibitory neuron synapses. Previous study has shown that inflammation induced loss of inhibitory nerve terminals or a redistribution of presynaptic machinery in inhibitory neurons may increase risk for developing neurological disease and psychiatric illness, including seizures and schizophrenia^39^.

Regarding genes involved in neuron function and plasticity, we continue to see universal downregulation in the flight tissue compared to GC controls. The gene SIN3A (https://www.ncbi.nlm.nih.gov/gene/?term=NM_001110350) behaves as a transcriptional repressor involved in cortical neuron differentiation^40^. In past studies the silencing of this gene in mice has shown decreased memory consolidation^41^. It is possible that downregulation may have similar, albeit reduced effects on mice in spaceflight. RELN (https://www.ncbi.nlm.nih.gov/gene/?term=NM_011261) codes for an extracellular matrix serine protease that regulates microtubule function and plays a role in the layering of neurons in the cerebral cortex, among other structures^42^. Its enzymatic activity that is important for cell adhesion and reduced transcription, as seen in our flight mice, could lead to further reduction of nervous system plasticity. SLC2A5 (https://www.ncbi.nlm.nih.gov/gene/?term=NM_019741) in a gene responsible for the GLUT5 (https://www.ncbi.nlm.nih.gov/gene/2700445) fructose transporter that has been found in numerous areas including the BBB, and the hippocampus^43,44^. The downregulation of this gene suggests the decreased ability of cells to utilize fructose as a potential energy source.

Level of gene expression related to neuron support cells, like microglial cells and oligodendrocytes were also seen universally downregulated. Microglia are the resident phagocytes of the innate immune system in the brain and play an important role in cytokine production^45^. Their activation is closely associated with environmental stress and microglial dysfunction is implicated in many neurological disorders and diseases^46^. CSF1R (https://www.ncbi.nlm.nih.gov/gene/?term=NM_001037859) is a gene coding for a cell-surface receptor which binds to colony stimulating factor-1 (CSF1) (https://www.ncbi.nlm.nih.gov/gene/?term=NM_001113529) and interleukin-34 (IL-34) (https://www.ncbi.nlm.nih.gov/gene/?term=NM_001135100)^47^. CSF1R null mice have been shown to have fewer phagocytic cells and reduced survival^48^. Given these findings we suggest that flight mice may have a reduced capacity to generate microglial cells in the nervous system. MERTK (https://www.ncbi.nlm.nih.gov/gene/?term=NM_008587) creates a kinase protein in innate immune cells which mediates engulfment of apoptotic cells and acts as a negative regulator of inflammation^49–51^. Downregulation of this gene may contribute to immune dysregulation in our flight mice. TREM2 (https://www.ncbi.nlm.nih.gov/gene/?term=NM_031254) codes for a regulator of microglial chemotaxis and phagocytosis of apoptotic^52,53^. In previous in vivo studies with microglial lacking TREM2, decreased migration to areas of apoptotic neurons was shown^54^. NCF1 (https://www.ncbi.nlm.nih.gov/gene/?term=NM_001286037) codes for a key subunit of the phagocyte NADPH oxidase system^55,56^. Downregulation suggests a reduced capacity for microglial cells to generate an effective microbial defense. SLC2A5 codes for the GLUT5 fructose transporter, as described previously^43^. With reduced activity, microglial cells may have less capacity to utilize fructose as an energy source and may be more prone to suffer from metabolic related dysfunction.

Oligodendrocytes are the myelin-forming cells of the CNS. It plays critical roles in axonal metabolic support, myelination, and providing nutritional support to neurons^57,58^. A couple of key genes involved in the oligodendrocyte function have been significantly downregulated in the flight mice. PLP1 (https://www.ncbi.nlm.nih.gov/gene/?term=NM_011123) codes for a major myelin protein that plays an important role in its formation and maintenance^59^. The implication of significantly downregulated PLP1 indicates impaired neuron axonal function with the possibility of long-term neurodegeneration. OPALIN (https://www.ncbi.nlm.nih.gov/gene/?term=NM_153520) plays a role in oligodendrocyte cytoskeletal remolding and morphology. Downregulation of this gene observed in our study may be an indication of decreased activity of oligodendrocytes. These findings are further reinforced by a significantly decreased oligodendrocyte function pathway score (Fig. 6a).

In terms of gene expression that associated with general immune functions and inflammation, only two genes were found to be significantly upregulated in our flight mice population, H2-T23 (https://www.ncbi.nlm.nih.gov/gene/?term=NM_010398) and SOX4 (https://www.ncbi.nlm.nih.gov/gene/?term=NM_009238). H2-T23 expression is essential for immunological protection and regulation^60^. HT-T23 dependent T-cell inhibitory interactions aid in preventing expansion of autoreactive CD4 T-cells and collateral autoimmune diseases^61^. Upregulation of this gene may be a protective response to generalized inflammation in the flight mice. SOX4 directly regulates Innate-like γδ T-cell (Tγδ17), a major source of interleukin-17 (IL-17) (https://www.ncbi.nlm.nih.gov/gene/?term=NM_010552). Increased SOX4 expression may lead to an increase of this cytokine expression in the flight mice^62^.

The gene RIPK1 (https://www.ncbi.nlm.nih.gov/gene/?term=NM_009068) has been shown to be a promotor of survival, apoptotic and inflammatory signaling pathways^63,64^. However, mice deficient in RIPK1 demonstrated loss of inhibition of a necroptosis pathway which promoted the release of necroptotic damage-associated molecular patterns^65^. Downregulation of RIPK1 observed in our study may lead to a similar reduction of appropriate apoptosis and an increase in necroptosis resulting in undesirable inflammation. Overall, taking these findings in conjunction with a significantly increased cytokine signaling pathway score (Fig. 6b), inflammatory signaling appears to be significantly dysregulated in our spaceflight mice.

Immune dysfunction is further illustrated via the downregulation of two genes important for antimicrobial defense. MPEG1 (https://www.ncbi.nlm.nih.gov/gene/?term=NM_010821) is a pore-forming bactericidal molecule of the innate immune system. It has been shown to be required for the activity of ROS and nitric oxide and their antibacterial effects^66,67^. OPTN (https://www.ncbi.nlm.nih.gov/gene/?term=NM_181848) codes for a protein that plays a key role in bacterial autophagic clearance. Deficient mice have also been shown to have impaired interferon regulatory factors (https://www.ncbi.nlm.nih.gov/gene/?term=NM_001159393) signaling and reduced response to toll-like receptor stimulation^68^. In summary, these results imply the impaired antibacterial capabilities and increased viral susceptibility in the flight mouse brain compared to GC group.

Our data indicate that the regulation of innate immune response was impaired following spaceflight. The pathway score for the innate immune response was significantly reduced in the flight group compared to GCs (Fig. 6c). The cell type score for exhausted CD8 cells is also greatly reduced for the flight group compared to controls and may indicate a dysfunctional phenotype in CD8 T-cell response. T-cell exhaustion represents an adaptive response to conditions of chronic antigen stimulation and inflammation^69^, as well as promoting tissue repair following an inflammatory injury^70^. Functionally exhausted CD8+ cells may result in a severely compromised innate immune response^71^.

Cytochrome P450 27A1 (CYP27A1) (https://www.ncbi.nlm.nih.gov/gene/?term=NM_024264) plays an important role in the metabolism of cholesterol and cholesterol-related compounds^72^. In humans, complete CYP27A1 deficiency leads to nodule formation in the brain which may lead to dementia, cerebellar ataxia, and spinal cord paresis^73^. This altered cholesterol metabolism closely associates with inflammatory responses involved in the pathogenesis of AD progression^74^. In deficient mice, a significant increase in cholestanol in the brain is observed^75^. Downregulation of CYP27A1, as seen in our flight mice, may lead to neurodegeneration and inflammation in more chronic settings or to a milder degree.

Fourteen genes that were significantly altered in the flight mice compared to GCs were found to have direct roles in cellular growth, proliferation, and stress response. Two upregulated genes play an important part in cell cycle arrest during times of cellular stress. GADD45G (https://www.ncbi.nlm.nih.gov/gene/?term=NM_011817) has been found to play a role in activating checkpoints in the cell cycle following exposure of cells to irradiation^76^. CCNG2 (https://www.ncbi.nlm.nih.gov/gene/?term=NM_007635) contributes to cell cycle arrest during DNA damage and is upregulated in response to diverse stimuli, including hypoxia^77^. Additionally, expression of CCNG2 is found to be significantly increased in cell cycle-arrested and terminally differentiated cells^78^. Upregulation of these genes may be due to exposure to irradiation, among other stressors, during spaceflight.

The downregulation of genes involved in promoting transcription and cell cycle progression was also seen. BRD2 (https://www.ncbi.nlm.nih.gov/gene/?term=NM_010238) codes for a nuclear kinase involved in regulating the expression of cell cycle genes via binding to multiple E2Fs, a family of transcription factors^79^. E2F1 (https://www.ncbi.nlm.nih.gov/gene/?term=NM_007891) regulates activation of DNA replication and G1/S transition when interacting with BRD2^80^. BRD2 also interacts with BRD3, a chromatin reader with roles in regulating transcription^81^. BRD3 (https://www.ncbi.nlm.nih.gov/gene/?term=NM_001113573) further regulates transcription by promoting the binding of the transcription factors to their targets^82^. SIN3A (https://www.ncbi.nlm.nih.gov/gene/?term=NM_001110350) codes for a transcription factor which regulates cell cycle progression by repressing gene expression for cell cycle inhibitor^40^. It has been linked to functional cellular changes in proliferation, cell cycle, and stem cell function in mice models^83^. Downregulation of these genes involved in the promotion of cellular proliferation may further amplify the upregulation of genes with roles in cell cycle arrest. These changes are similarly reinforced by a significantly increased epigenetic regulation pathway score (Fig. 6e) indicating that there may be an increased level of modulating and checks being implemented during transcription, due to spaceflight. A recent study has shown that cell cycle regulation is closely linked to hippocampal neurogenesis which plays critical roles in memory and learning^84^. Neurogenesis is a dynamic process that involves proliferation and differentiation of stem and progenitor cells, or survival and maturation of newborn neurons^84^. Environmental stressors have been shown to modulate cell cycle progression, particular in the G1 phase^85^. These observed changes of gene expression related to the cell cycle in our study may indicate altered regulation of neurogenesis.

Many downregulated genes were known to be involved in cellular stress responses. MAP2K4 (https://www.ncbi.nlm.nih.gov/gene/?term=NM_009157) is a kinase involved with responses to cellular stress including apoptosis, inflammation, cellular proliferation, and neurodegeneration. A deficiency of MAP2K4 has been shown to result in cellular susceptibility to stress-induced apoptosis and growth inhibition^86^. However, it has been demonstrated that disruption of both MAP2K4 and MAP2K7 (https://www.ncbi.nlm.nih.gov/gene/?term=NM_001042557) genes was required to thoroughly block cellular growth caused by environmental stressors^87^. MRE11A (https://www.ncbi.nlm.nih.gov/gene/?term=NM_018736), a part of an exonuclease complex central to the cellular DNA damage response was downregulated. The MRE11 complex is essential to vertebrates and defects lead to sensitivity to DNA damage and cell cycle checkpoints deficiency^88^. Its downregulation may point to a certain level of dysfunction leading to an impaired response to cellular stress leaving the flight mice more vulnerable to DNA damage.

Different brain regions have different vulnerability to environmental stressors due to structural and biological heterogeneity of the regions, cell types and molecular networks^89^. Regional differences of protein biomarkers in response to stressors were observed in a previous flight study^41^. Given this finding, gene expression profiles in specific regions of the brain in response to spaceflight will be investigated in future studies. More recently, single-cell sequencing technologies, including transcriptomics, are available by directly measuring multiple molecular signatures in specific brain cells, providing robust molecular identity of specific cell types^90^.

Brain tissues were dissected and prepared for analysis within 60 h after landing. It is possible that observed changes in gene expression profiles in neuroinflammation are a compensatory acute response to the acceleration and intense noise of launch or reflect changes to the combined response of spaceflight environment and the landing. In order to evaluate the immune response to spaceflight environment, in the further study, mice will be euthanized in the orbit and brain tissues will be immediately preserved for analysis on the ground. Furthermore, in order to test the hypothesis that neuroinflammation plays an important role in developing spaceflight-induced stress response in the brain, an anti-oxidant or anti-inflammatory compound will be injected to mouse before or during the flight to determine the protective effect against oxidative damage in the brain.

It is noted from our study that among those genes which expression were significantly altered by spaceflight, markedly more gene expressions are being downregulated than upregulated. It is speculated that many genes may be upregulated during spaceflight and the early phase after landing as an adaptation response. During re-adaptation, the expression of these previous upregulated genes could be then downregulated as a compensatory response, or when protein synthesis from upregulated genes has been completed, these genes expression may be downregulated^32^.

Collectively, changes noted in our study indicate that exposure to the spaceflight environment induces significant changes in gene expression and signaling pathways related to neuronal function, immune regulation, growth and metabolic function. Study showed that chronically dysfunctional and deregulated pathways, including cytokine signaling (Fig. 6b), epigenetic regulation (Fig. 6e), and notch signaling (Fig. 6f) play important roles in disease development^91^, and have been associated with several progressive neurodegenerative diseases^92^. Our observed changes of a dysregulated inflammatory response, downregulated T-cell response, and reduced microglia signaling (Fig. 6g) might also have an impact on brain structure and function, and further lead to chronic neuroinflammation. It has been shown that here are strong correlations between neuroinflammatory biomarkers, brain morphology and behavioral outcomes^93^. It may be hypothesized that chronical changes of observed gene expression profiles could have long-term effects on brain morphology and organism behavior.

## Materials and methods

### Flight and ground control conditions

Ten-weeks-old male C57BL/6 mice (Jackson Laboratories, Inc. Bar Harbor, ME) at the time of launch, were flown for NASA’s ninth Rodent Research experiment (RR-9) on SpaceX-12 for a 35-day mission and lived in NASA’s Rodent Habitats aboard the International Space Station. All FLT mice were maintained at an ambient temperature of 26–28 °C with a 12-h light/dark cycle during the flight. Habitat GC mice were kept under similar housing conditions, including temperature, humidity and carbon dioxide (CO_2_) levels using 48-h delayed telemetry data from the FLT group. Water and food bar diet specifically designed by NASA were provided ad libitum to FLT and GC groups. All mice received the same access to food and water. NASA-Ames Research Center and KSC Institutional Animal Care and Use Committees approved this flight study. The study has been done in strict accordance with the recommendations in the Guide for the Care and Use of Laboratory Animals of the National Institute of Health.

### Mouse brain dissection after spaceflight

Within 38 ± 4 h of splashdown, the FLT mice were rapidly euthanized in 100% CO_2_. The GC mice were euthanized with the same method after 38 days of GC housing. Shortly after euthanasia, brains were removed and bisected along the midline and coronally within the half hemispheres. The right caudal half hemisphere of the brain (containing mid- and hindbrain) from each mouse (*n* = 5–6 per group) was placed in a sterile cryovial, snap frozen in liquid nitrogen and kept at −80 °C prior to use.

### RNA isolation and gene expression profiling

Isolation of total RNA from the brain tissues was performed using DNA/RNA/miRNA Universal Kit (Qiagen, # 80224) according to the manufacturer’s instruction. Briefly, tissue homogenization was performed using 1.5 mm beads (Benchmark, # 1032-15) on Minilys homogenizer (Bertin Technologies) in RLT Plus buffer. The purity and concentration of the eluted RNA were measured using Nanodrop 2000 (Thermo Fisher Scientific, Waltham, MA) and stored at −80 °C until further analysis. RNA samples (20 ng/ul) were then shipped to Nanostring (Technologies Seattle, WA) and gene expression profiling of brain tissues was conducted using the nCounter^®^ neuroflammation pathways panel. The panel includes 757 genes covering the core pathways and processes that define neuroimmune interactions and 13 potential housekeeping genes for normalization. RNA samples (100 ng each) on 2 cartridges were used for the Gene Expression Assay with Mouse Neuroinflammation panel performed on the nCounter MAX system (https://nanostring.com/wp-content/uploads/MAN-C0035_nCounter_Analysis_System_MAX_FLEX.pdf), a multi-channel epifluorescence scanner with Nanostring Advanced Analysis Module plugin for QC, normalization, and differential expression analysis (DE). Data files generated from nCounter system were analyzed using nSolver 4.0 software with the Advanced Analysis module for QC (quality control), normalization, DE analysis, and gene-set enrichment analysis. Data normalization included 2 steps: Positive control normalization to correct platform-associated variation and Codeset content normalization using the housekeeping (HK) genes to correct variability of input samples. Geometric means of selected HK probes were used to normalize counts of the samples and DEGs were generated from the normalized counts.

### Statistical analysis

Gene expression profiling data were analyzed using nSolver analysis and the advanced analysis module software. Advanced analysis module software uses open-source R program for pathway scoring and gene-set enrichment analysis. Using the Reactome pathway database annotations, pathway scores are derived by calculating the first principle component of pathway genes’ normalized expression^86^ and data are summarized from changes of a gene set within a given pathway into a single score. To further analyze the associated pathways of the DEGs, Kyoto Encyclopedia of Genes and Genomes (KEGG) pathways analysis was performed using biological process database to identify the annotated sets of genes based on the biological processes in which they participate. Significantly differential expression genes were presented in the tables with individual genes using log_10_ (*p* value) andlog_2_ fold change compared to the GC group. Pathview module was used to display upregulated genes or downregulated genes overlaid on KEGG pathways. For DEG and pathway analysis, *p* < 0.05 was considered statistically significant between FLT and GC groups by one-way ANOVA and Tukey’s HSD test.

### Reporting summary

Further information on research design is available in the Nature Research Reporting Summary linked to this article.

## Supplementary information

Dataset

Reporting Summary

## Data Availability

The authors declare that source data supporting the findings of this study with the figures of the article are provided with this paper.

## Code availability

All of the significant gene data obtained in this study were deposited in the NCBI’s GEO, and are accessible through GEO accession number GSE186278.
