# Supplementary material for: Characterization of gene expression profiles in the mouse brain after 35 days of spaceflight mission
Source: NPJ Microgravity. 2022 Aug 10;8:35. doi: 10.1038/s41526-022-00217-4 (PMC9365836; doi:10.1038/s41526-022-00217-4)
Supplement: Supplementary file 2 — Reporting Summary [file 41526_2022_217_MOESM2_ESM.pdf]

nature portfolio

Corresponding author(s): Xiao Wen Mao

Last updated by author(s): Jun 28, 2022

Reporting Summary

Nature Portfolio wishes to improve the reproducibility of the work that we publish. This form provides structure for consistency and transparency in reporting. For further information on Nature Portfolio policies, see our Editorial Policies and the Editorial Policy Checklist.

Statistics

For all statistical analyses, confirm that the following items are present in the figure legend, table legend, main text, or Methods section.

n/a	Confirmed	
☐	☒	The exact sample size (n) for each experimental group/condition, given as a discrete number and unit of measurement
☐	☒	A statement on whether measurements were taken from distinct samples or whether the same sample was measured repeatedly
☐	☒	The statistical test(s) used AND whether they are one- or two-sided<br>*Only common tests should be described solely by name; describe more complex techniques in the Methods section.*
☐	☒	A description of all covariates tested
☐	☒	A description of any assumptions or corrections, such as tests of normality and adjustment for multiple comparisons
☐	☒	A full description of the statistical parameters including central tendency (e.g. means) or other basic estimates (e.g. regression coefficient) AND variation (e.g. standard deviation) or associated estimates of uncertainty (e.g. confidence intervals)
☐	☒	For null hypothesis testing, the test statistic (e.g. F, t, r) with confidence intervals, effect sizes, degrees of freedom and P value noted<br>*Give P values as exact values whenever suitable.*
☒	☐	For Bayesian analysis, information on the choice of priors and Markov chain Monte Carlo settings
☒	☐	For hierarchical and complex designs, identification of the appropriate level for tests and full reporting of outcomes
☒	☐	Estimates of effect sizes (e.g. Cohen's d, Pearson's r), indicating how they were calculated

Our web collection on statistics for biologists contains articles on many of the points above.

Software and code

Policy information about availability of computer code

Data collection	Gene Expression Assay with Mouse Neuroinflammation panel performed on the nCounter MAX system (https://nanostring.com/wp-content/uploads/MAN-C0035_nCounter_Analysis_System_MAX_FLEX.pdf), a multi-channel epifluorescence scanner with Nanostring Advanced Analysis Module plugin for QC, normalization, and differential expression analysis (DE).
Data analysis	Data files generated from nCounter system were analyzed using nSolver 4.0 software with the Advanced Analysis module for QC (quality control), normalization, DE analysis, and gene-set enrichment analysis.

For manuscripts utilizing custom algorithms or software that are central to the research but not yet described in published literature, software must be made available to editors and reviewers. We strongly encourage code deposition in a community repository (e.g. GitHub). See the Nature Portfolio guidelines for submitting code & software for further information.

Data

Policy information about availability of data

All manuscripts must include a data availability statement. This statement should provide the following information, where applicable:

- Accession codes, unique identifiers, or web links for publicly available datasets
- A description of any restrictions on data availability
- For clinical datasets or third party data, please ensure that the statement adheres to our policy

The authors declare that source data supporting the findings of this study with the figures of the article are provided with this paper.

March 2021

Field-specific reporting

Please select the one below that is the best fit for your research. If you are not sure, read the appropriate sections before making your selection.

☒ Life sciences ☐ Behavioural & social sciences ☐ Ecological, evolutionary & environmental sciences

For a reference copy of the document with all sections, see nature.com/documents/nr-reporting-summary-flat.pdf

Life sciences study design

All studies must disclose on these points even when the disclosure is negative.

Sample size	there were 6 sample per group
Data exclusions	Data from one mouse in flight group is exclused from pathway scoring calculation as outlier determined by calculating upper boundary and lower boundary by taking 3 standard deviation from the mean of the values
Replication	This is a spaceflight study. We only have access to this one flight. This experiment can not be replicated.
Randomization	The images were taken randomly over the sections.
Blinding	The investigators were blinded during data collection and analysis.

Reporting for specific materials, systems and methods

We require information from authors about some types of materials, experimental systems and methods used in many studies. Here, indicate whether each material, system or method listed is relevant to your study. If you are not sure if a list item applies to your research, read the appropriate section before selecting a response.

Materials & experimental systems

n/a	Involved in the study	
☒	☐	Antibodies
☒	☐	Eukaryotic cell lines
☒	☐	Palaeontology and archaeology
☐	☒	Animals and other organisms
☒	☐	Human research participants
☒	☐	Clinical data
☒	☐	Dual use research of concern

Methods

n/a	Involved in the study	
☒	☐	ChIP-seq
☒	☐	Flow cytometry
☒	☐	MRI-based neuroimaging

Animals and other organisms

Policy information about studies involving animals; ARRIVE guidelines recommended for reporting animal research

Laboratory animals	10 week old male C57BL/6 mice ordered from Jackson lab were used.
Wild animals	Our study does not involve wild animals.
Field-collected samples	Our study does not involve field-collected samples
Ethics oversight	The study has been done in strict accordance with the recommendations in the Guide for the Care and Use of Laboratory Animals of the National Institute of Health (NIH). The protocol was approved by NASA FICUC and Loma Linda University IACUC.

Note that full information on the approval of the study protocol must also be provided in the manuscript.
